# Protocol to study synapse density or volume—SynDOVE—in brain using confocal microscopy and Imaris three-dimensional surface rendering software

**DOI:** 10.1016/j.xpro.2026.104465

**Published:** 2026-04-02

**Authors:** Nolwazi Z. Gcwensa, Khaliah Y. Long, Arielle F. Manabat, Laura A. Volpicelli-Daley

**Affiliations:** 1 Killion Center for Neurodegeneration and Experimental Therapeutics, University of Alabama at Birmingham, Birmingham, AL 35294, USA

**Keywords:** Health Sciences, Microscopy, Cognitive Neuroscience

## Abstract

We present a protocol for analyzing pre- and postsynaptic loci in fixed brain sections using confocal microscopy images and Imaris software. We describe steps for capturing z stack images of immunofluorescent-labeled synaptic proteins using a confocal microscope. We then detail procedures for deconvolving images, rendering three-dimensional (3D) surface recontructions of synaptic markers, and isolating closely juxtaposed pre- and post-synaptic 3D surfaces, herein termed “synaptic loci.” This protocol allows for quantitation of synaptic loci density, as well as pre- and postsynaptic volumes.

For complete details on the use and execution of this protocol, please refer to Gcwensa et al.^1^

## Highlights

• Guidance on navigating Imaris interface for synapse density and volume analysis
• Protocol for rendering three-dimensional surfaces of synaptic proteins
• Step-by-step guide to isolate synaptic loci and generate appropriate statistics
• Procedure for calculating density of synaptic loci and volume of puncta

## Abstract (editor highlights)

Publisher’s note: Undertaking any experimental protocol requires adherence to local institutional guidelines for laboratory safety and ethics.

## Graphical abstract

**Figure:**
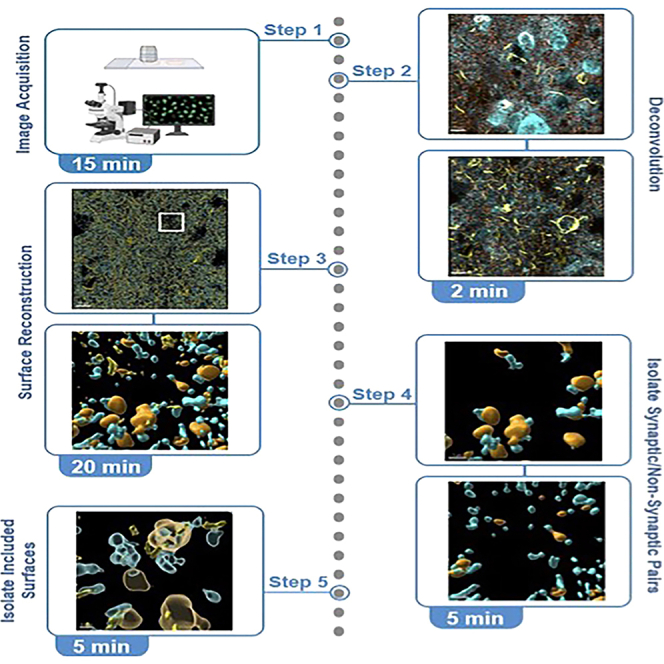

## Teaser

We present a protocol for analyzing pre- and postsynaptic loci in fixed brain sections using confocal microscopy images and Imaris software. We describe steps for capturing z stack images of immunofluorescent-labeled synaptic proteins using a confocal microscope. We then detail procedures for deconvolving images, rendering three-dimensional (3D) surface reconstructions of synaptic markers, and isolating closely juxtaposed pre- and post-synaptic 3D surfaces, herein termed “synaptic loci.” This protocol allows for quantitation of synaptic loci density, as well as pre- and post-synaptic volumes.

## Before you begin

### Innovation

Synaptic dysfunction is a hallmark of numerous neurological disorders including but not limited to autism, schizophrenia, mood disorders, and neurodegenerative diseases. Being able to quantify this synaptic dysfunction is critical. However, pre-existing methods are limited to two-dimensional analyses or rely on costly super-resolution imaging techniques, which require specialized training and restrictive tissue preparation protocols. This protocol describes a cost-effective, reproducible, and technically accessible way to analyze synaptic structures *in situ* in brain tissue that does not require super-resolution imaging or additional training. We outline step-by-step how to navigate the Imaris software interface to generate pre- and post-synaptically juxtaposed puncta, or synaptic loci. Second, we provide detailed guidance on how to appropriately analyze the mean density and volume of total puncta and synaptic loci. This protocol can be applied, but not limited, to understanding synaptic biology and the study of neurological disease mechanisms.

### Turn on the confocal microscope

**Timing: 10 min**

For the purposes of this protocol, we have detailed image acquisition and image processing steps. Please note that we use a Nikon A1R microscope and software for image acquisition and deconvolution, but other confocal microscopes and similar deconvolution software can be used. For example, imaging parameters (e.g., laser settings, optical gain, z-stack) can vary based on experimenter’s needs and can be optimized using different confocal microscopes, in such that the experimenter should not be limited by the following example instructions using a Nikon A1R microscope.

1. Ensure objectives and imaging slides are thoroughly cleaned.
2. Mount slide (see problem 2 in ‘troubleshooting’ section for potential challenges).
3. Apply appropriate immersion material for selected objective.
4. Navigate to region to be imaged (*see note*).
  a. Select ‘eyepiece’ - EPI for eye port mode.
  b. Navigate to region of interest (ROI) using epifluorescence light source and oculars.
    ***Note:*** Avoid regions that have artefacts (i.e. blood vessels, torn tissue, uneven staining, mounting media bubbles) that may impact the signal and therefore the surface rendering when comparing different images. Please refer to problem 4 in the “troubleshooting” section for potential challenges.
  c. Use low magnification air objectives to identify ROI.
  d. Use high magnification oil objectives to focus microscope on ROI.
5. Collect images in highest magnification for microscope (see problem 3 in ‘troubleshooting’ section for potential challenges).
  a. Select 60X 1.4 NA plan apochromatic oil objective.
  b. Add immersion oil to objective.

### Set up imaging parameters on a confocal microscope

**Timing: 5–10 min per image**

6. Select Nikon C2 for camera mode.
7. Set lasers and ‘gain’ to desired parameters for each channel (*see note*).
  a. Optimize laser and gain settings based on control sample and keep constant across all samples within experiment.

***Note:*** Independent experiments may require adjustments in laser and gain settings. Experimenter should optimize laser and gain settings to meet the following criteria: Look-up table (LUT) histogram values are no greater than approximately ½ total histogram value, minimal to no oversaturated pixels within collection range, and signal-to-noise allows clear distinction between puncta and background signal by eye.

8. Setup tissue depth for z-stack (see problem 8 in ‘troubleshooting’ section for potential challenges).
  a. Set middle of the z-stack to the midpoint of clearest signal determined in camera mode.
    i. Step: 0.125 μm (*see note*).
    ii. Range: 2 μm (*see note*).

***Note:*** Selecting range for z stack can vary and needs to be optimized by investigators for each experiment. Tissue penetration of different antibodies can also vary. It is recommended to collect images within the middle-most section of total thickness of signal penetration where all antibodies are equally distributed throughout the tissue. Additionally, it is important that investigators adhere to the recommended Nyquist Z resolution values of 0.1–0.2 μm when acquiring images. The z stack should range from 20 to 25 images at a thickness (or depth) of 1-3 μm. For additional guidance on challenges due to imaging parameters, please refer to problem 8 in the ‘troubleshooting’ section.

### Deconvolve images

**Timing: ∼1–2 min per image**

Here, NIS Elements Imaging Software (or similar software) can be used for image deconvolution using the Richardson-Lucy algorithm (*see note*).

9. Using NIS BatchDeconvolution 6.10.
10. Load z-stack images to be deconvolved by selecting ‘add file’ or ‘add folder’.
11. Select ‘Next’.
12. Select method: Richardson-Lucy.
13. Select noise level: clear.
14. Select iterations: 20.

***Note:*** The choice for deconvolution method and number of iterations is best determined by investigators based on the needs of the analysis. More iterations will increase the time taken for deconvolution and may not necessarily improve the quality of the outcome images. Additionally, while this protocol provides instruction using NIS BatchDeconvolution software, other software that can use the Richardson-Lucy algorithm include but are not necessarily limited to DeconvolutionLab, AutoQuant, Imaris ClearView-GPU Deconvolution, Deconwolf, Zeiss Deconvolution toolkit, Leica deconvolution, and MATLAB.

15. Select ‘Finish’ to deconvolve the images.
16. Save deconvolved images (*see note*).

***Note:*** Most of the parameters will be automatically updated into the software based on the meta data from the image if the image is saved as an ND2 file. If the parameters are not automatically uploaded, investigators may need to store the meta data and manually upload values into the deconvolution software. Please refer to problem 5 in the ‘troubleshooting’ section for potential challenges regarding image quality.

### Imaris surface reconstruction

Before you begin surface reconstruction, it is important to familiarize yourself with the Imaris interface. Though user friendly, the system can be confusing for first time users. It is encouraged for users of this protocol to begin by accessing the numerous resources from Oxford Instruments, including Imaris tutorials (https://imaris.oxinst.com/tutorials), to learn how to navigate Imaris. If user cannot access the Imaris software, please refer to problem 7 in the ‘troubleshooting’ section.

### Navigating the Imaris interface

**Figure 1:**
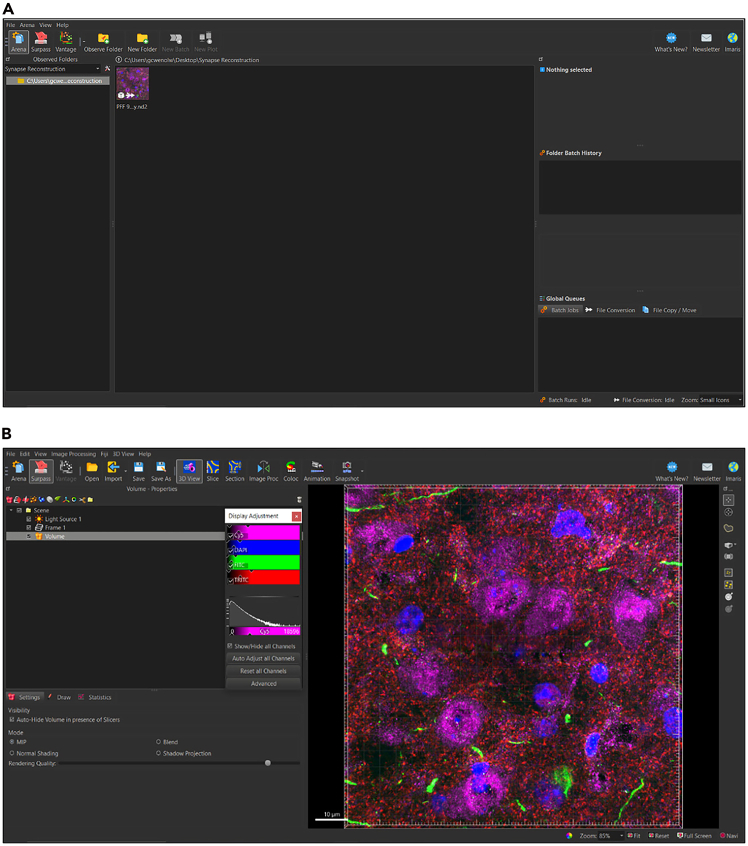
Distinguishing between ‘area view’ and ‘surpass view’ in Imaris (A) Image of Arena View in Imaris which allows users to convert files into IMS format and navigate between different image files. (B) Image of Surpass View in Imaris which allows users to manipulate individual images and add different objects including surface volumes.

Before the user navigates the Imaris interface, the Arena View versus the Surpass View in the Imaris software needs to be distinguished. The Arena View allows the user to navigate through different image files (Figure 1A), whereas the Surpass View allows the user to manipulate different objects, such as surfaces, on individual images (Figure 1B).

### In Arena View

In Arena View (Figure 1A), users can toggle between different observation folders to view any Imaris compatible image files, such as ND2 files acquired from a confocal microscope. Here, users are also able to convert image files into the IMS file format necessary for image manipulation in the Imaris software.

### Institutional permissions

The Institutional Animal Care and Use Committee at the University of Alabama at Birmingham approved all animal protocols were performed in accordance with regulatory standards. For details on animal care, please refer Nolwazi Z Gcwensa et al.^1^ Readers are advised to refer to their institution-specific guidelines and acquire permission from relevant institutions.

## Key resources table

**Table:** 

REAGENT or RESOURCE	SOURCE	IDENTIFIER
**Software and algorithms**		

NIS Elements AR 6.10.01	Nikon Instruments Inc.	RRID: SCR_014329
Imaris	Bitplane	RRID:SCR_007370

**Antibodies**		

VGLUT2	Synaptic Systems	135 421
HOMER1	Synaptic Systems	160 006
pSer129-synuclein	Abcam	51253

**Other**		

Nikon A1R Microscope	Nikon Instruments Inc.	N/A

***Note:*** A Nikon confocal microscope and associated image acquisition and image deconvolution software are not essential. Others can be used based on experimenter’s needs and availability. For example, images can successfully be obtained using a Crest Cicero Spinning Disk confocal, rather than a Nikon.

## Materials and equipment

**Table:** 

Antibody target	RRID	Host species/Isotype/Mono or Polyclonal	Dilution
VGLUT2	AB_2619823	Mouse/IgG/Mono	1 : 1 000
HOMER1	AB_2631222	Chicken/IgG/Poly	1 : 1 000
pSer129-synuclein	AB_869973	Rabbit/IgG/Mono	1 : 1 000

## Step-by-step method details

### Convert images to IMS format for Imaris

**Timing: 30 s per image**

Before adding Imaris objects to an image, the image must first be converted into IMS format.

**Figure 2:**
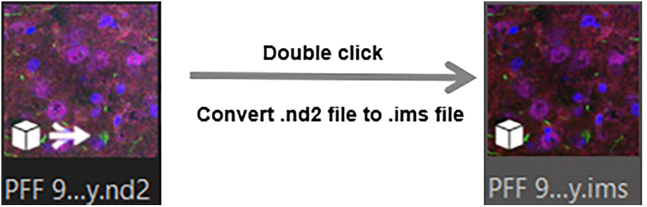
Conversion of ND2 files to IMS files Double-click image to automatically convert files to.ims format for Imaris. ND2 files will have the white 3D box and right-pointing arrow icons in the lower left corner. IMS files will have the 3D box icon in the lower left corner.

***Note:*** The Imaris software can import confocal images from multiple sources including but not limited to the following: TIFF, Leica, Zeiss, Olympus, and Nikon. Each of the corresponding image file formats can successfully be converted to Nikon ND2 format.

1. Hover the mouse over the image of interest.
2. Double-click image of interest (*see note*).
  a. ND2 confocal image will be converted to IMS Imaris image (see problem 1 in ‘troubleshooting’ section for potential challenges).
3. Confirm image conversion by noting the icons in the lower left corner of the image (Figure 2).
  a. ND2 files will have a white 3D box and a white right-pointing arrow.
  b. After double clicking on the image, the icon in the lower left should be a white 3D box only.

### Reset LUTs to normal levels

**Timing: 10 s**

In Surpass View (Figure 1B), users can adjust LUTs, view individual channels, and add Imaris objects, such as surfaces, to IMS files. Upon opening the Surpass View, users should find a 3D box containing the channels collected during image acquisition. Imaris will automatically enhance LUTs to maximize visibility of pixels. However, rendering surfaces using images with enhanced LUTs will affect thresholding and accuracy in the surface rendering. We suggest that users adjust LUTs to normal levels for all channels before beginning surface rendering.

**Figure 3:**
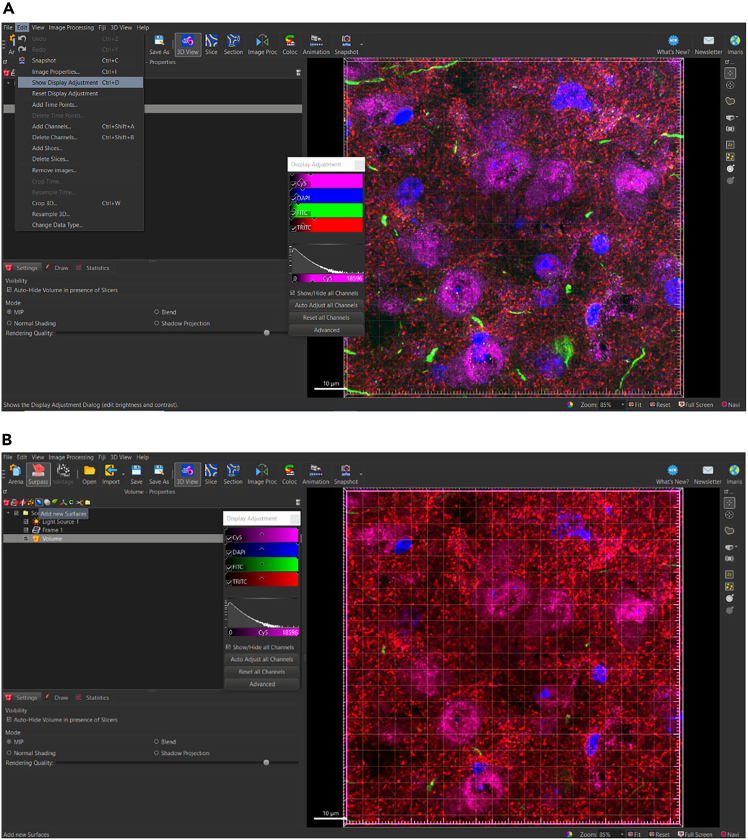
Resetting LUTs to normal levels Use Display Adjustment tab to reset LUTs to normal levels. (A) Image with LUTs automatically enhanced by Imaris. (B) Image with LUTs adjusted to normal levels.

4. Show display adjustment (Figure 3).
  a. Edit > Show Display Adjustment (Ctrl + D).
  b. Reset all channels.

### Render total volume surface

**Timing: 2 min**

**Figure 4:**
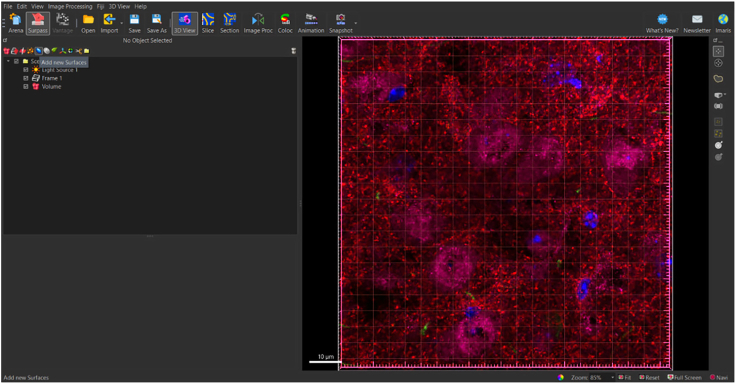
Adding new surface object for total volume In surpass view, select ‘Add new Surfaces’ icon to add new icon to Scene. All new items added to Scene are listed in order of addition.

To begin analyzing surfaces, the total number of puncta needs to be normalized to the total volume of the image. This is done to accommodate differences that may be observed due to potential image variability within each z-stack. To determine the volume of the 3D, box that contains all the puncta within the total z-stack, a 3D surface is rendered around the total z-stack as follows (Figure 4).

**Figure 5:**
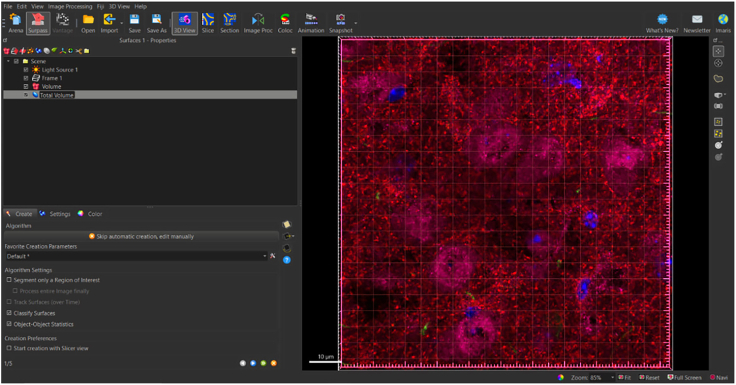
Changing title of new surface to ‘Total Volume’ Ensure that all surface object titles names are changed to appropriate new titles for clarity.

***Note:*** Make sure “Total Volume” surface is selected to open “Create” tab.

5. Generate new surface creation parameter for total volume surface (*see note*).
  a. Add new surface.
  b. Rename surface to ‘Total Volume’ (Figure 5).

6. Select ‘Create’ Tab: Algorithm.
7. De-select ‘Classify Surfaces’ (*see note*).

**Figure 6:**
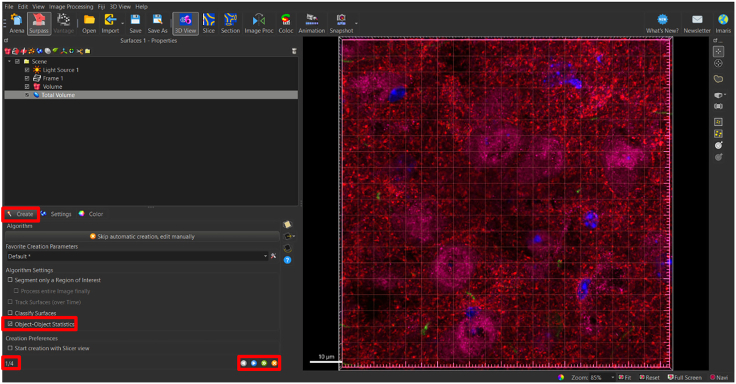
Navigating the surface creation tab The creation tab is indicated by a wand icon. Algorithm settings to note are indicated within a red box. Algorithm settings can be selected per the experimenters’ preferences, however, for statistical analyses to be performed, Object-Object Statistics’ must be selected in Algorithm settings. Selection and deselection of Algorithm settings can adjust the number of creation panels indicated in the bottom left. To toggle between creation panels, select the blue left/right arrows on the bottom right.

***Note:*** In this step, the software will automatically select ‘Object-Object Statistics’ and ‘Classify Surfaces.’ It is only necessary to de-select ‘Classify Surfaces.’ This is essential, because if ‘Object-Object Statistics’ is de-selected, it will disrupt the software’s ability to correctly generate data (denoted as ‘statistics’ in the Imaris software’ based on surfaces generated) (Figure 6).

8. Select blue right-pointing arrow to go to ‘Create: Segmentation Setup’ (Figure 6).
9. Select ‘Create’ Tab: Segmentation Setup.

**Figure 7:**
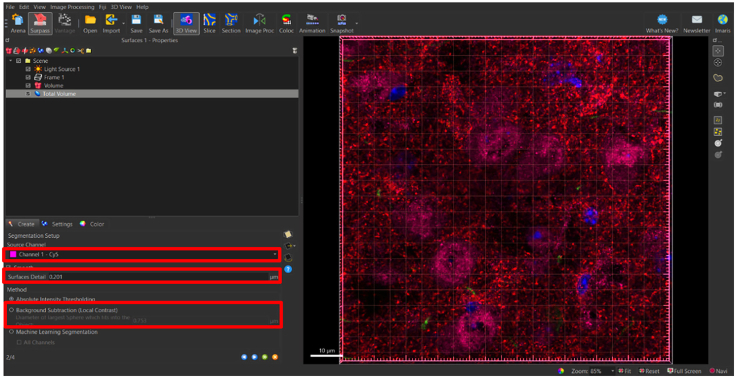
Setting up surface parameter details based on puncta diameter in segmentation setup Select channel surface and input values for surface detail and seed point diameter in this panel.

**CRITICAL:** Do not change settings (Figure 7).

**Figure 8:**
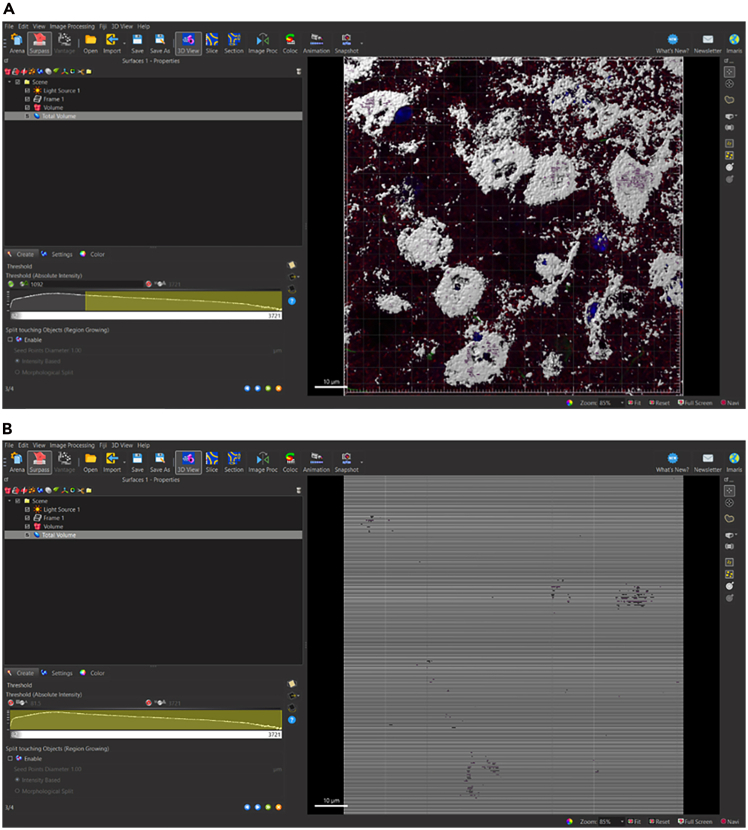
Selecting the level of signal threshold in threshold panel (A) Signal threshold can be set according to automatic values chosen by Imaris software or set manually by experimenter. (B) To generate surface volume for total volume of z-stack, drag yellow lower threshold value to the leftmost side of the histogram.

10. Select blue right-pointing arrow to go to ‘Create: Threshold’.
11. Select ‘Create’ Tab: Threshold.
12. Drag yellow lower threshold value to the leftmost side of the histogram (Figure 8B).

**CRITICAL:** Alternatively, insert the value “0” into the leftmost Lower Threshold (Figure 8A).

13. Select blue right-pointing arrow to go to ‘Create: Filter Surfaces’.
14. Select ‘Create’ Tab: Filter Surfaces.

**CRITICAL:** Do not change settings.

**Figure 9:**
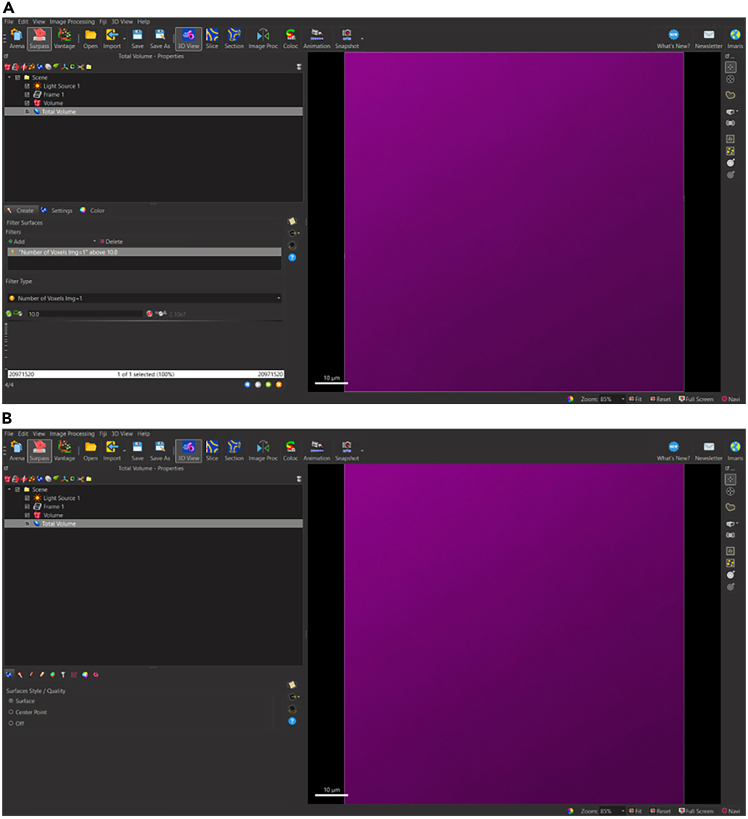
Completing Total Volume surface reconstruction (A) Select green right arrow to skip remaining Create parameters. (B) Complete surface rendering for Total Volume of z-stack.

15. Select green right-pointing double arrow to ‘Finish’ and execute all creation parameters (Figure 9A).

### Render surface volume for synaptic puncta

**Timing: 5 min**

Once the total volume surface has been rendered, the generated 3D surface specific to each synaptic channel can begin. The process of the surface creation is similar with a few adjustments made under each creation tab. Please reference the above images as we navigate through the different tabs under the Create tab. As an example, surfaces for the synaptic protein, vesicular glutamate transmitter 2 (VGLUT2), will be generated.

16. Display normal LUTs for VGLUT2 channel only.
  a. Show display adjustment.
    i. Edit > Show Display Adjustment (Ctrl + D).
    ii. Reset all channels.
    iii. Deselect all channels except VGLUT2 channel.
17. Generate new surface creation parameters for VGLUT2 surface.
  a. Add new surface.
  b. Rename surface to ‘VGLUT2’.
  c. Select ‘VGLUT2’ surface in order open ‘Create’ tab.
  d. Select ‘Create’ tab: algorithm.
  e. Deselect ‘Classify Surfaces’.
  f. Select blue right-pointing arrow to go to ‘Create: Segmentation Setup’.
    **CRITICAL:** Up to this point, all instructions have been regarding absolute outcomes. Hereafter, instructions will be adjustable depending on the needs of the user and the desired outcomes for the experiment. We suggest the following parameters with experiment-specific optimization. However, some alternative approaches for each parameter may affect the experimental outcome. Please refer to the instructions in detail and try alternative parameters and settings BEFORE finalizing parameters to be used in your own experiments.
  g. Select ‘Create’ tab: Segmentation Setup.
  h. Select ‘Source Channel’ drop down.
  i. Choose VGLUT2 source channel.
    i. Make sure the box next to ‘Smooth’ is selected.
  j. Select ‘Background Subtraction (Local Contrast)’, located under method.
  k. Determine value for ‘Diameter for largest sphere which fits into Object’.
    i. In ‘Slice’ view.
    ii. Scroll through z-stack using slider on the left (Figure 10A).

**Figure 10:**
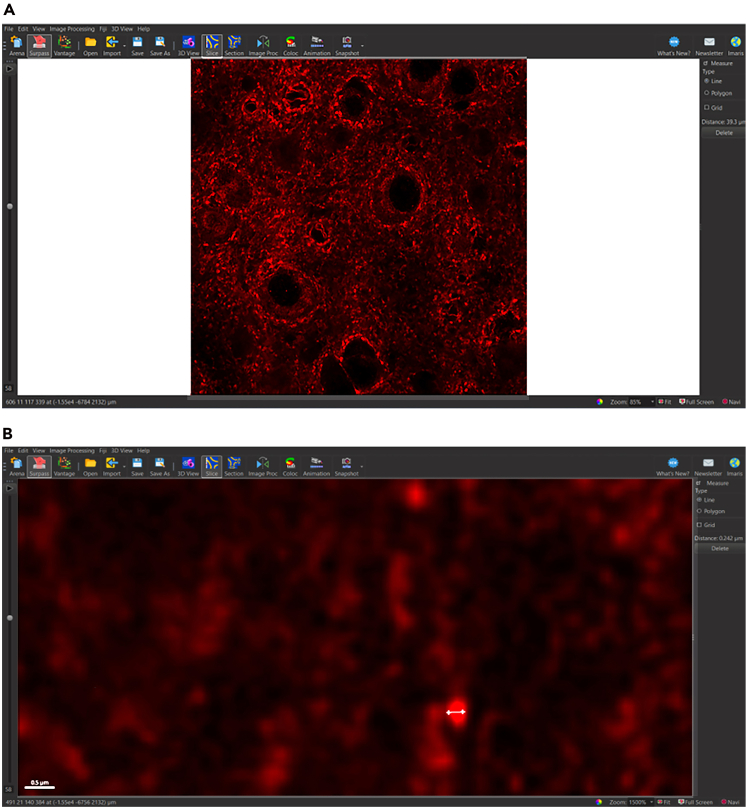
Determining the smallest diameter of puncta (A) In slice view, toggle between individual images within the z-stack to inspect individual puncta determined to be true signal. (B) Magnify puncta of interest and create a line representing the diameter by clicking on either side of a number of small puncta. Average line measurement from several puncta for approximate estimate of diameter of smallest puncta. Ensure line is measured along the same axis for all selected small puncta.
    iii. Select smallest puncta determined to be in-focus signal.
    iv. Click on either side of the puncta to determine diameter length (Figure 10B).
    v. Record diameter length indicated in right-most panel in slice view.
    vi. Repeat this for a few puncta (along the same axis) to determine approximate value for the average diameter of the smallest puncta.
    vii. Return to ‘3D View’.
    viii. Input average diameter determined above into ‘Diameter for largest sphere which fits into Object’: 0.270 μm (Figure 7).
      **CRITICAL:** To ensure the segmentation operates above the optical resolution limit of the imaging system, the recommended value for ‘Diameter of Largest Sphere’ was set to approximately 2**–**3 times the lateral (x-y) pixel width. For confocal images acquired at 1024 x 1024 x-y resolution under Nyquist sampling conditions, the lateral voxel size was approximately 0.1 μm as determined through image metadata, corresponding to a minimal puncta diameter of 0.170–0.350 μm.
18. Determine surface detail.
  a. Input value that is ∼half of ‘Diameter for largest sphere which fits into Object’ value determined above: 0.140 μm (Figure 7).
19. Select blue right-pointing arrow to go to ‘Create: Threshold’.
20. Select ‘Create’ tab: Threshold.
21. Determine lower ‘Threshold (Background Subtraction)’ value according to the following equation (Figure 11, *see note*):
  a. $\%\;\left(\text{lower}\;\text{threshold}\right)=\frac{\text{Imaris}\;\text{Automatic}\;\text{Value}\;}{\text{Maximum}\;\text{Threshold}\;\text{Value}}\times 100$.
  b. Percentage lower threshold = $\frac{273\;}{2730}\times 100=10\;\%$.

**Figure 11:**
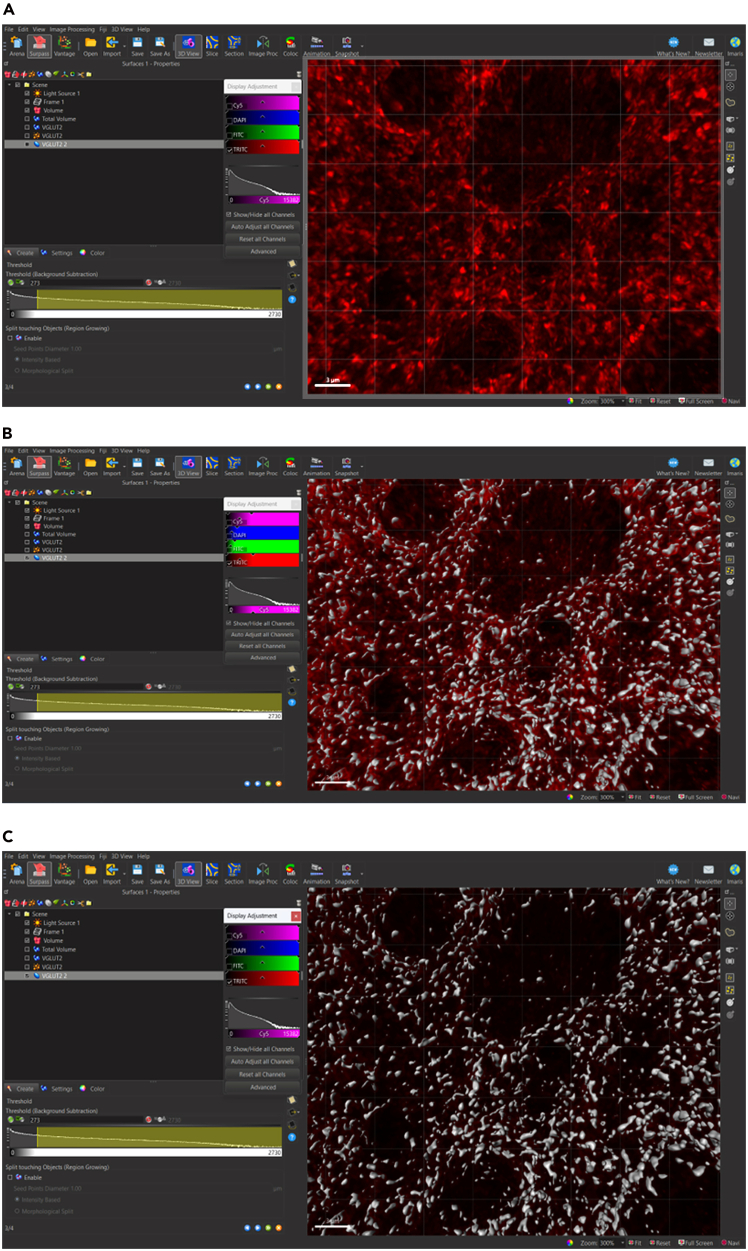
Setting signal threshold for surface reconstruction (A) Toggle VGLUT2 surface objects ‘off’ with LUTs adjusted to true signal levels to visualize image signal. (B) Toggle VGLUT2 surface objects ‘on’ with LUTs set to auto-adjust to show misleading signal enhancement. (C) Toggle VGLUT2 surface objects ‘on’ with LUTs set to true signal levels to show partial volume rendering encompasses all true signal values.
22. Determine the Percentage Lower threshold for multiple images across multiple tissue samples including control tissue samples.
23. Determine the average value for Percentage Lower Threshold across all experimental samples.
24. Input the Percentage Lower Threshold Value for VGLUT2 to be used across all VGLUT2 surface rendering if all parameters are kept constant.
25. Use visual inspection by magnifying image and toggling surface on and off to determine if creation parameters are sufficiently capturing all signals.
26. Select blue right-pointing arrow to go to ‘Create: Filter Surfaces’.

***Note:*** The synaptic threshold, or background subtraction, is determined using the global average of percent lower threshold values across all treatment groups. Calculating the percent lower threshold is outlined in Step 21a and 21b. It is important to note that this process will need to be repeated for each protein surface that will be generated. After completing this process for synaptic proteins, we suggest a Percentage Lower Threshold between 5-10% of the Maximum Threshold Value (Step 23) is sufficient in adequately capturing signal. To further establish the specific value that best represents signals in a sample, it is suggested that investigators use visual inspection outlined in Step 25.

27. Select ‘Create’ tab: Filter Surfaces.
28. Remove surfaces resulting from aberrations (*see note*).
  ***Note:*** Imaris adds filter: Number of voxels Img = 1 above 10.0.
  a. Magnify area with low density of surfaces.
  b. Toggle ‘Number of voxels’ filter and surface on and off to visually determine level of minimum voxel size compared to signal.
  c. Add Filter: Volume (Figure 12).
    i. Magnify area with low density of surfaces.
    ii. Toggle ‘Volume’ filter and surface on and off to visually determine lower and/or upper threshold value for surface volumes compared to signal:
    iii. Lower (left) threshold: deactivated.
    iv. Upper (right) threshold: 2.00 μm^3^.

**Figure 12:**
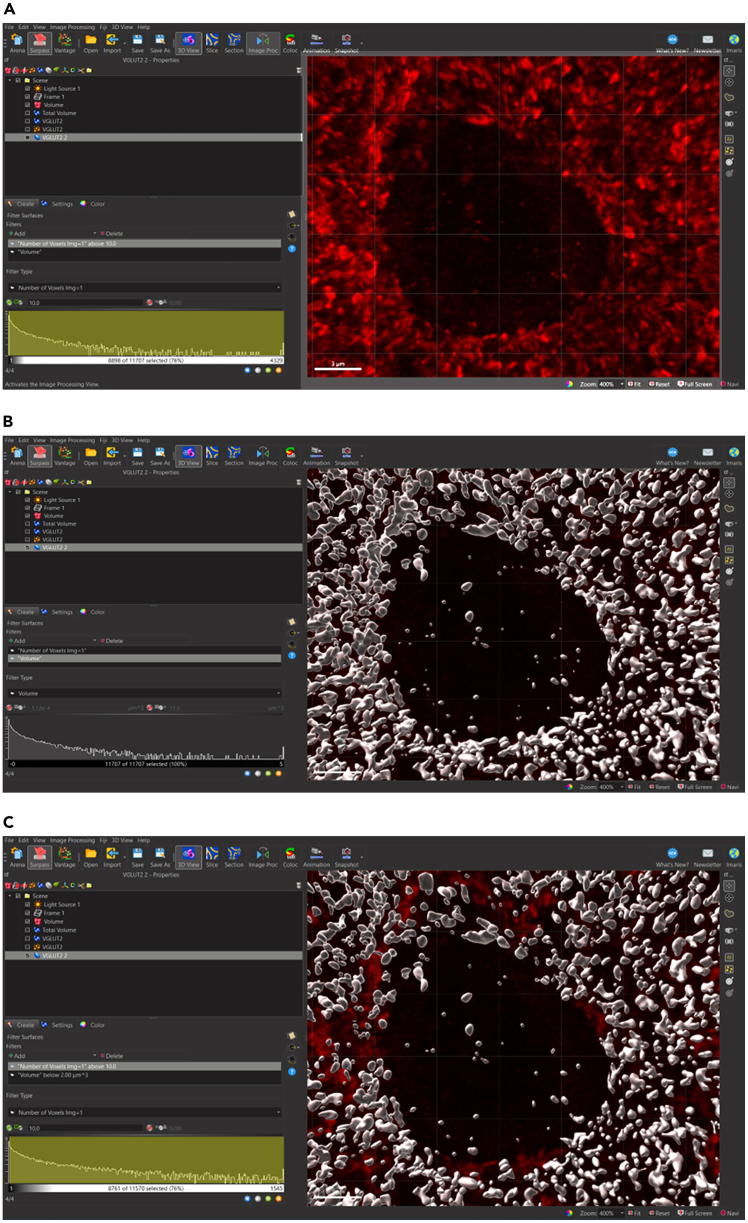
Filtering surfaces to remove surface rendering artefacts (A) Toggle volume ‘off’ to visualize signal with no LUT adjustment. (B) toggle volume ‘on’ with all filters deactivated to visualize surfaces based in previous parameters. (C) Toggle volume ‘on’ with voxel and upper volume threshold activated to visualize final surfaces to be rendered.
29. Select green right-pointing double arrow to ‘Finish’ and execute all creation parameters.
30. Store creation parameters to apply to all images in experiment (*see note*, Figure 13)*.*
  a. Select VGLUT2 surface.
  b. Select ‘Creation’ wand icon.
  c. Select ‘Store Parameters for Batch’ tab.
  d. Store parameters in favorite creation parameters for future reference.

**Figure 13:**
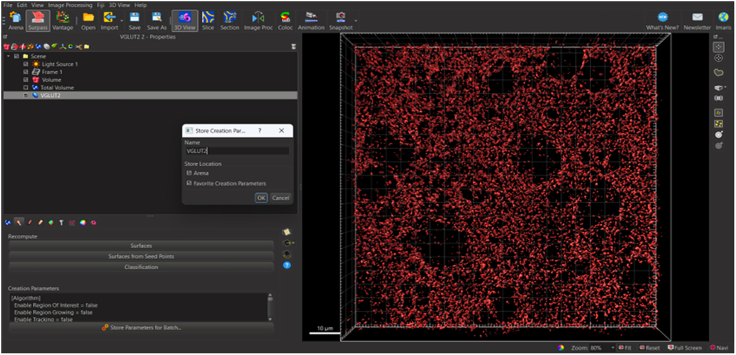
Storing surface parameters for application to remaining images in experiment With desired surface selected, click creation wand and select ‘Store Parameters for Batch’ tab to store surface parameters in Arena or Favorite Creation Parameters drop down menu.

***Note:*** With appropriate adjustments, these parameters can be used for punctate and globular/filamentous proteins, such as p-α-synuclein. However, for proteins with defined nuclei and projections such as astrocytes and microglia, consider using other Imaris functions like ‘Filaments’.

### Isolate surfaces of synaptic proteins that are closely juxtaposed—Synaptic loci

**Timing: 10 min**

Once the surfaces for at least two synaptic proteins have been rendered, proteins that are closely localized can be isolated to determine pre- and postsynaptically juxtaposed puncta, termed ‘Synaptic Loci’. In this image rendering we have rendered surfaces for pre-synaptic protein, VGLUT2 and post-synaptic protein, HOMER1.

**Figure 14:**
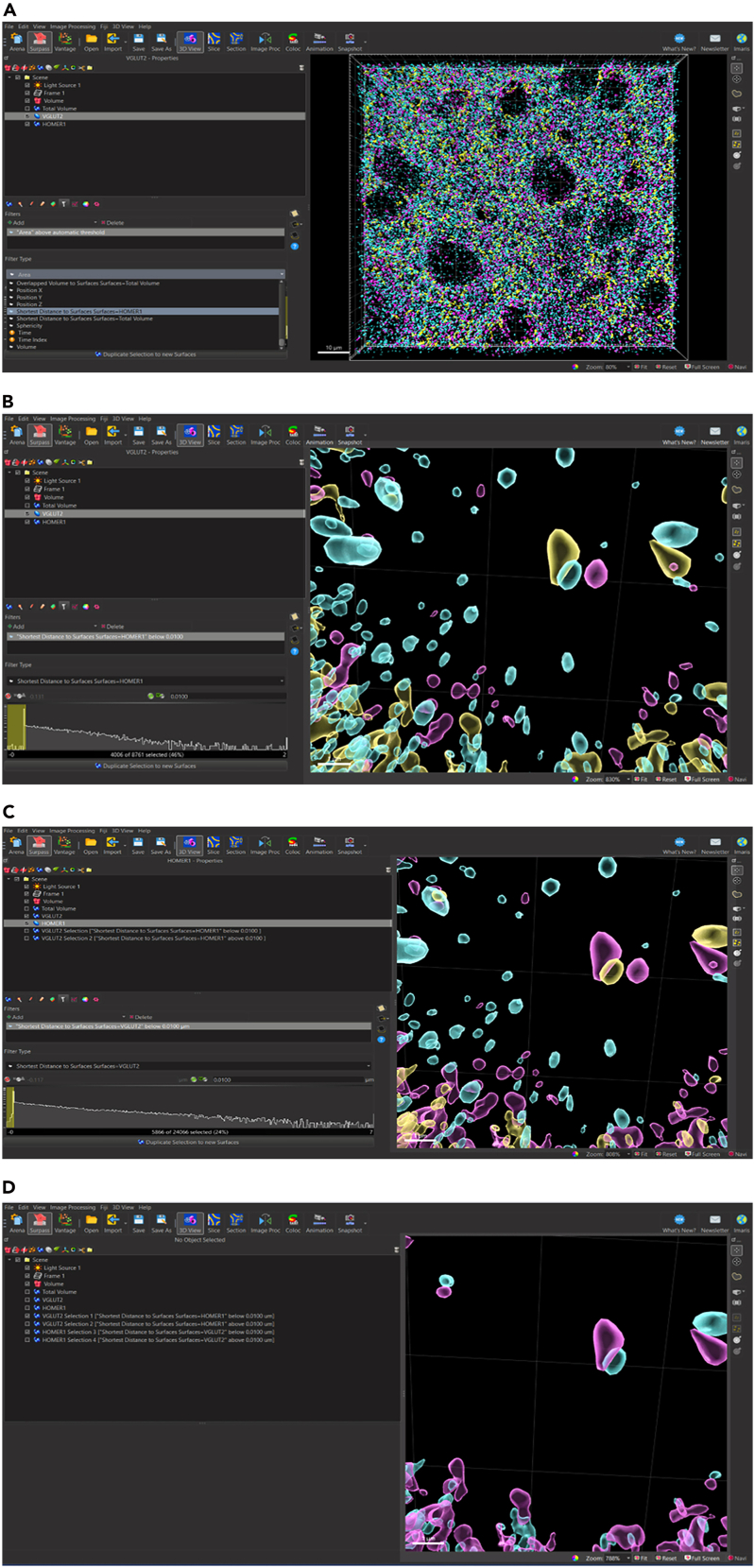
Filtering surfaces to generate synaptic loci – surfaces closely juxtaposed to corresponding synaptic surfaces (A) Select desired synaptic surface VGLUT2 to activate, select filter icon, select drop down menu, and select ‘Shortest Distance to Surface=HOMER1’ as filter parameter. (B) For VGLUT2 surfaces (magenta) closely juxtaposed to HOMER1 surfaces (cyan), activate right thresholding and set to manual threshold 0.01 μm. For unpaired surfaces, activate left thresholding and set to manual threshold 0.01 μm. (C) For HOMER1 surfaces (cyan) relative to VGLUT2 surfaces (magenta) repeat filter parameter setting as in (B). Activated surfaces which fall within the filter parameter are indicated in yellow. (D) HOMER1 surfaces (cyan) and VGLUT2 surfaces (magenta) closely juxtaposed to corresponding synaptic surfaces are defined as synaptic loci.

**Figure 15:**
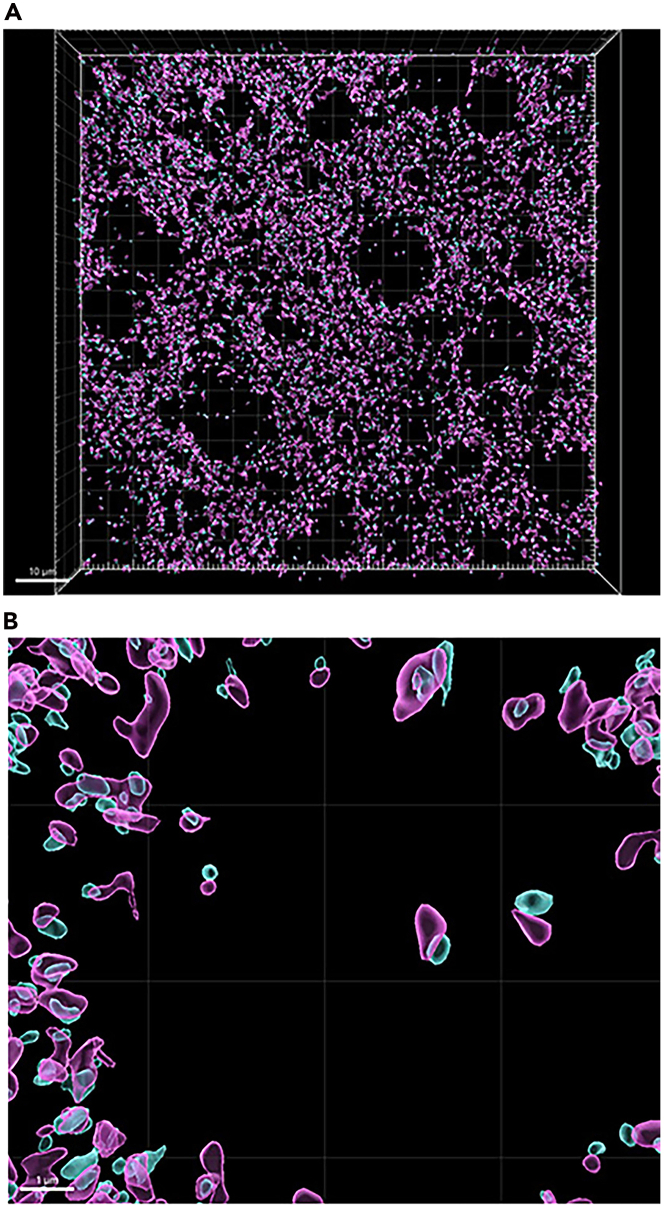
Synaptic loci isolated from synaptic surfaces not closely juxtaposed to corresponding synaptic surface (A) Zoomed out view of VGLUT2 (magenta) surfaces and HOMER1 (cyan) surfaces closely juxtaposed to corresponding synaptic surfaces indicated by white arrowheads. Scale bar = 10 μm. (B) Zoomed in view of VGLUT2 (magenta) surfaces and HOMER1 (cyan) surfaces closely juxtaposed to corresponding synaptic surfaces. Scale bar = 1 μm.

31. Filter to separate VGLUT2 surfaces closely juxtaposed to HOMER1 surfaces.
  a. Select VGLUT2 surfaces to activate surface.
  b. Select ‘Filter’ tab.
  c. Active VGLUT2 surfaces appear yellow.
  d. Select Filter icon to add filters.
  e. Select ‘+ Add’ Filter.
  f. Navigate to ‘Filter Type’ Menu.
  g. Select drop down arrow.
  h. Scroll down to select ‘Shortest Distance to Surfaces Surfaces=HOMER1’.
  i. Set shortest distance threshold.
    i. Lower (left) Threshold: Deactivate; value: N/A.
    ii. Upper (right) Threshold: Activate; value: 0.01 μm (*see note*)*.*
      ***Note:*** The 0.01 μm shortest distance threshold in Step 31i, ii was chosen based on the structure of the synapse, in which electron microscopy studies have estimated central synapses can range roughly between 12 and 30 nm.^2^^,^^3^ Please refer to problem 6 in the ‘troubleshooting’ section for additional guidance.
  j. Select ‘Duplicate Selection to new Surfaces” (*see note*)*.*
    ***Note:*** The ‘Shortest Distance to Surfaces=HOMER1’ filter option will not appear if original surface creation parameter ‘Object-Object Statistics’ was not selected in first page of surface creation parameter ‘Create” Algorithm”.
32. Filter to separate VGLUT2 surfaces NOT closely juxtaposed to HOMER1 surfaces.
  a. Select VGLUT2 surfaces to activate surface.
  b. Repeat steps 31a-i.
  c. Set shortest distance threshold.
    i. Lower (left) Threshold: Activate; value: N/A.
    ii. Upper (right) Threshold: deactivate; value: 0.01 μm.
  d. Select ‘Duplicate Selection to new Surfaces”.
33. Filter to separate HOMER1 surfaces closely juxtaposed to VLUT2 surfaces.
  a. Select HOMER1 surfaces to activate surface.
  b. Repeat all the above steps 31a-i for HOMER1 surfaces closely juxtaposed to VGLUT2 and not closely juxtaposed to VGLUT2 Figures 14 and 15.

### Generate surfaces for proteins within or near the surfaces of synaptic proteins

**Timing: 5 min**

While pre- and post-synaptic proteins are expected to render closely juxtaposed surfaces, occasionally there may be a need to render and isolate surfaces for proteins which are expected to be localized within or near synapses. The above procedure for rendering synaptic proteins and isolating closely juxtaposed surfaces can be easily adjusted to render and isolate surfaces for other nearby proteins. Examples of proteins of interest near or within synapses could include but are not limited to phosphorylated α-synuclein (p-α-syn), phosphorylated tau, β-amyloid, TDP-43, cytochrome C, and C1q. To demonstrate how this protocol can be further adapted, an example for pre-synaptic VGLUT2, post-synaptic HOMER1, and p-α-syn is used below, since p-α-syn inclusions can be observed within pre- and post-synaptic surfaces.

34. To generate new surface creation parameters for p-α-syn surface.
  a. Complete surface reconstruction for ‘total volume’, VGLUT2′ and ‘HOMER1’.
  b. Repeat all the above Steps 17-29 to render surfaces for p-α-syn. Refer to Table 1 for parameters used for surfaces rendering protocol.

**Table 1:** Example surface parameters for surfaces rendered

Surface	Surface grain size (μm)	Diameter of largest sphere (μm)	Threshold value (% of total signal)	Filter surfaces: Voxels	Filter surfaces: Volume (μm3)
Total Volume	Automatic Value	Automatic Value	100	N/A	N/A
VGLUT2	0.200	0.400	10	>10	N/A
HOMER1	0.150	0.300	10	>10	<2.00
pSer129-syn	0.05	0.6	2	N/A	N/A

### Isolate synaptic proteins with p-α-syn inclusions from synaptic proteins without p-α-syn inclusions

**Timing: 2 min**

**Figure 16:**
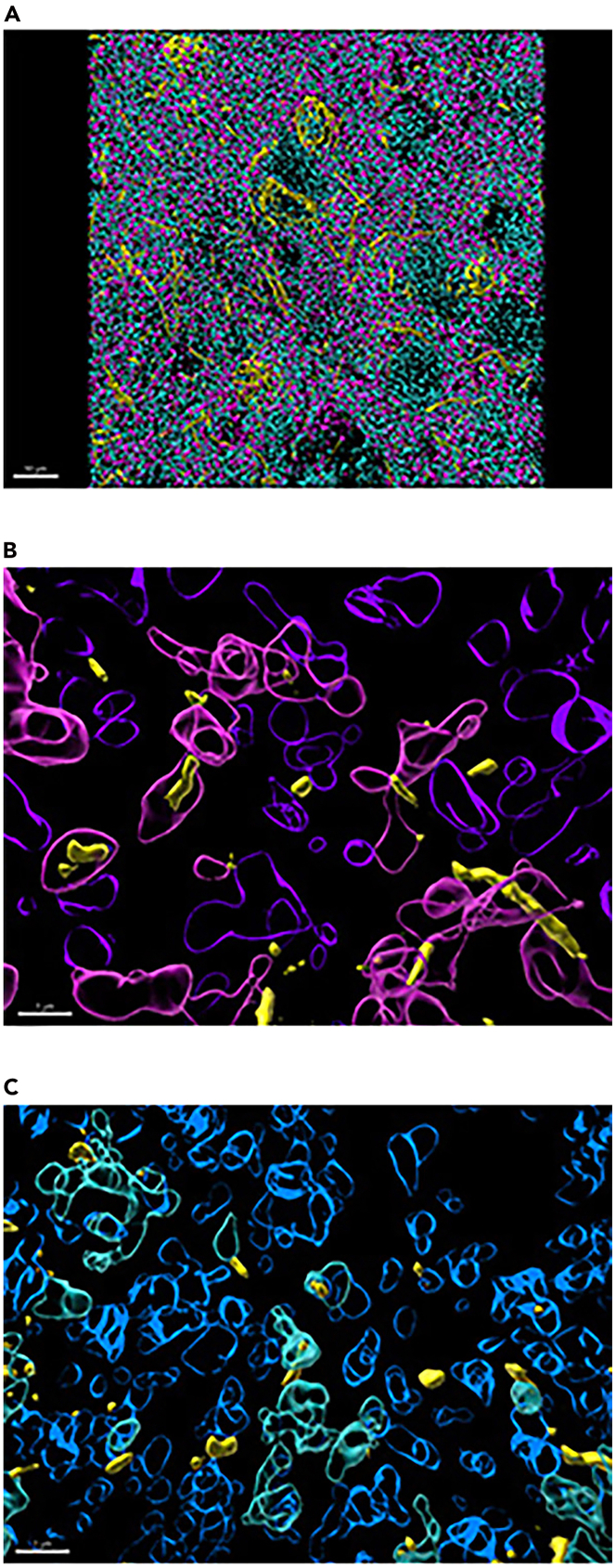
Surfaces rendered for synaptic proteins and p-α-synuclein inclusions (A) Total surfaces rendered for VGLUT2 (magenta), HOMER1 (cyan) and p-α-syn (yellow). Scale bar = 10 μm. (B) p-α-syn inclusions (yellow), VGLUT2 containing p-α-syn inclusions (magenta, white arrowhead), VGLUT2 surfaces NOT containing p-α-syn inclusions (purple, white arrow). Scale bar = 1 μm. (C) p-α-syn inclusions (yellow), HOMER1 containing p-α-syn inclusions (cyan, white arrowhead), HOMER1 surfaces NOT containing p-α-syn inclusions (blue, white arrow). Scale bar = 1 μm.

35. Filter to isolate p-α-syn surfaces included within VGLUT2+ surfaces.
  a. Select VGLUT2 surfaces to activate surface.
  b. Select ‘Filter’ tab.
  c. In ‘Filter’ tab, add filter.
    i. Select ‘+ Add’ Filter.
  d. Under ‘Filter Type’ Menu, select filter type.
    i. Select drop down arrow.
    ii. Scroll down to select ‘Shortest Distance to Surfaces Surfaces=p-α-syn.
  e. Set shortest distance threshold as follows:
    i. Lower (left) Threshold: Deactivate; value: N/A.
    ii. Upper (right) Threshold: Activate; value: 0.1 μm.
  f. Select ‘Duplicate Selection to new Surfaces”.
36. Filter to isolate p-α-syn surfaces NOT within VGLUT2+ surfaces.
  a. Select VGLUT2 surfaces to activate surface.
  b. Select ‘Filter’ tab.
  c. In ‘Filter’ tab, add filter.
    i. Select ‘+ Add’ Filter.
  d. Under ‘Filter Type’ Menu, select filter type.
    i. Select drop down arrow.
    ii. Scroll down to select ‘Shortest Distance to Surfaces Surfaces=p-α-syn.
  e. Set shortest distance threshold.
    i. Lower (left) Threshold: Activate; value: 0.1 μm.
    ii. Upper (right) Threshold: Deactivate; value: N/A.
  f. Select ‘Duplicate Selection to new Surfaces”.
37. Filter to isolate p-α-syn surfaces included within HOMER1+ surfaces (Figure 16).
  a. Repeat Steps 35a-f to generate the corresponding surfaces for HOMER1 surfaces containing p-α-syn and not containing p-α-syn.

### Storing data for analysis

**Timing: 2 min**

**Figure 17:**
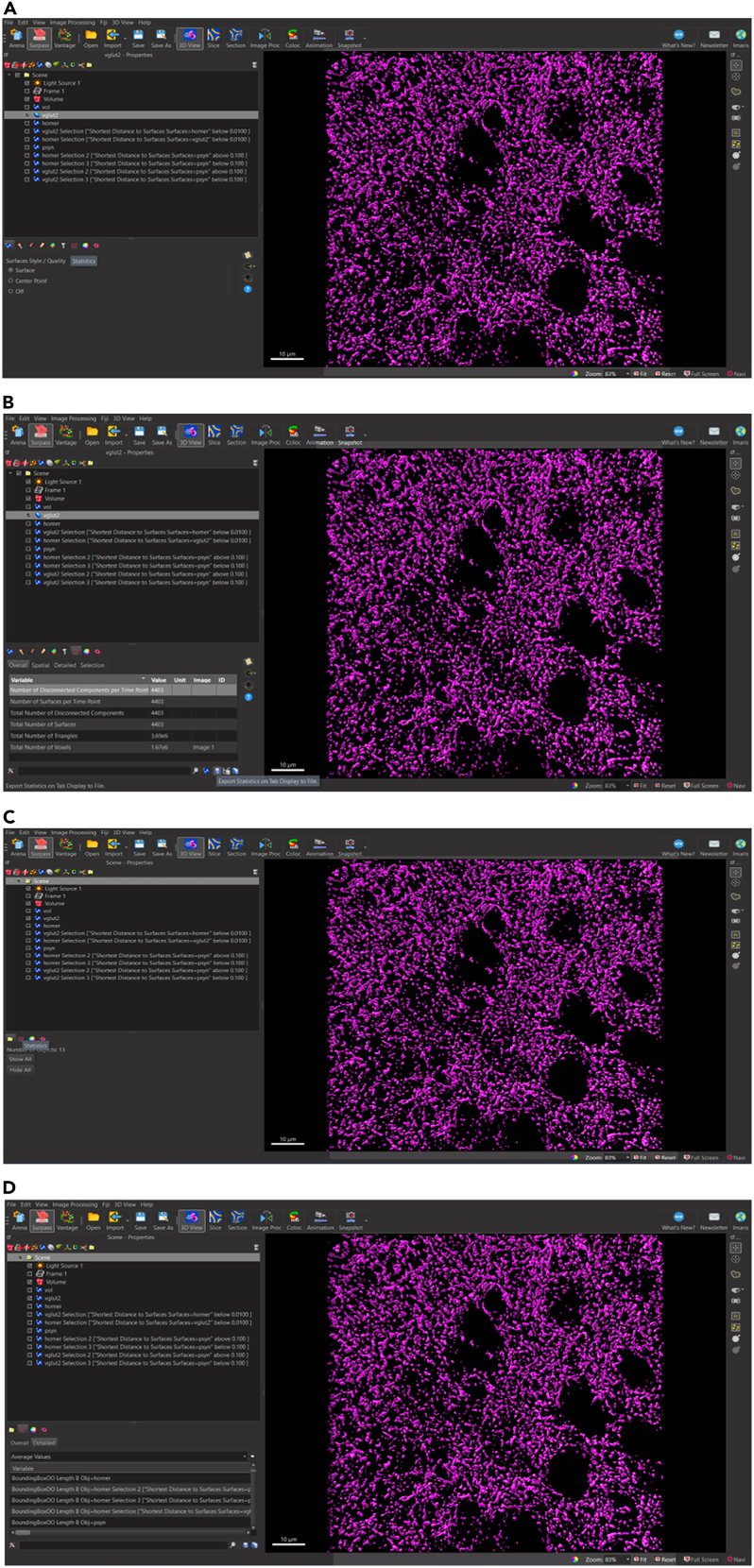
Storing desired surface data (denoted as ‘statistics’ in the Imaris software) for further analysis (A) Store data for individual surfaces by selecting surface of choice, VGLUT2 (B) Select ‘Statistics’ graph icons and select save icon to ‘Export Statistics on Tabs Display to File’. To store data for all surface objects (C) Select ‘Scene’ folder then select ‘Statistics’ graphs icon (D) Select ‘Detailed’ tab and select ‘Average Values’ in drop down menu to save mean values for all selected surface statistics.

At this point, surfaces have been generated for ‘total volume,’ which refers to the volume containing all protein-specific surface objects, as well as surfaces for VGLUT2+ pre-synaptic surfaces, HOMER1+ post-synaptic surfaces, p-α-syn+ surfaces, and the VGLUT2/HOMER1+ surfaces which are closely juxtaposed (e.g., considered synaptic loci) and those that are ± p-α-syn inclusions. Due to the number of surfaces generated throughout this protocol, it is critical that all surfaces are appropriately named to minimize risk of confusion or mistakes during statistical analysis. In the following instructions, it is shown how to create and store the data generated by Imaris for each surface, with ‘data’ often denoted as ‘statistics’ in the Imaris software. Saving ‘mean statistics,’ as denoted in the Imaris software, for each surface object will be demonstrated (Figure 17).

**CRITICAL:** Please be aware that filtered surfaces, e.g. VGLUT2 filtered for juxtaposition to HOMER1 surfaces, have a naming system generated by Imaris based on filter parameters used and may not be intuitively understood. These may need to be changed accordingly.

38. To generate individual data files for generated surface object: VGLUT2.
  a. Activate surface VGLUT2.
  b. Select ‘Statistics’ tab.
  c. Select ‘Overall’ tab (*see note*).
  d. Select Save icon (Figure 15).
  e. Select Single save icon: Export Statistics on Tab Display to File.
    ***Note:*** This stores the data or ‘statistics’ selected in the tab only (in this case ‘Overall Statistics’).
  f. Save file as.csv, .xml or .xls as desired.
  g. Repeat Step 38 a-g for all surface objects desired for further analysis (*see note*)*.*
    ***Note:*** Storing data for individual surface objects allows you to store data for each surface rendered (e.g., on the VGLUT2 object).
39. Generate ‘mean statistics’ for ALL generated surface objects.
  a. Activate surface ‘Scene’ by selecting the folder at the top of the list of objects (*see note*)*.*
    ***Note:*** It does not matter which surfaces are activated or inactivated. Selecting Scene applies all instructions to every object generated on the open Imaris file.
  b. Select ‘Statistics’ tab.
  c. Select ‘Detailed’ Tab.
  d. Select ‘Average Values’ in drop down menu.
  e. Select Save icon (Figure 15).
  f. Select single save icon: Export Statistics on Tab Display to File.
  g. Save file as .csv, .xml or .xls as desired.
  h. Repeat these steps for any and all surface objects desired for further analysis (*see note*)*.*
    ***Note:*** Considering that most experiments will have multiple images for multiple animals and multiple treatments, this can result in statistical analyses encompassing potentially innumerable data points. We recommend spending time devising a statistical strategy before determining which data or ‘statistics’ files to store from Imaris.

## Expected outcomes

Several quantitative metrics can be captured from Imaris when performing this protocol in immunofluorescent-labelled tissue. Figure 14 shows the expected outcome for surface reconstructions for pre- and post-synaptic markers, VGLUT2 and HOMER1, as well as inclusion protein, p-α-syn. Imaris can create data or ‘statistics’ tables containing numerical values for each surface object representing the volume, count, surface area, and overlapped volume ratio to surface X. Shortest distance to surface X can also be acquired. The values can be compared between surface objects in the same image (i.e., synaptic VGLUT2 volume vs unpaired VGLUT2 volume) or compared between experimental treatments (i.e., mean volume of VGLUT2 in treated vs non-treated animals).

**Figure 18:**
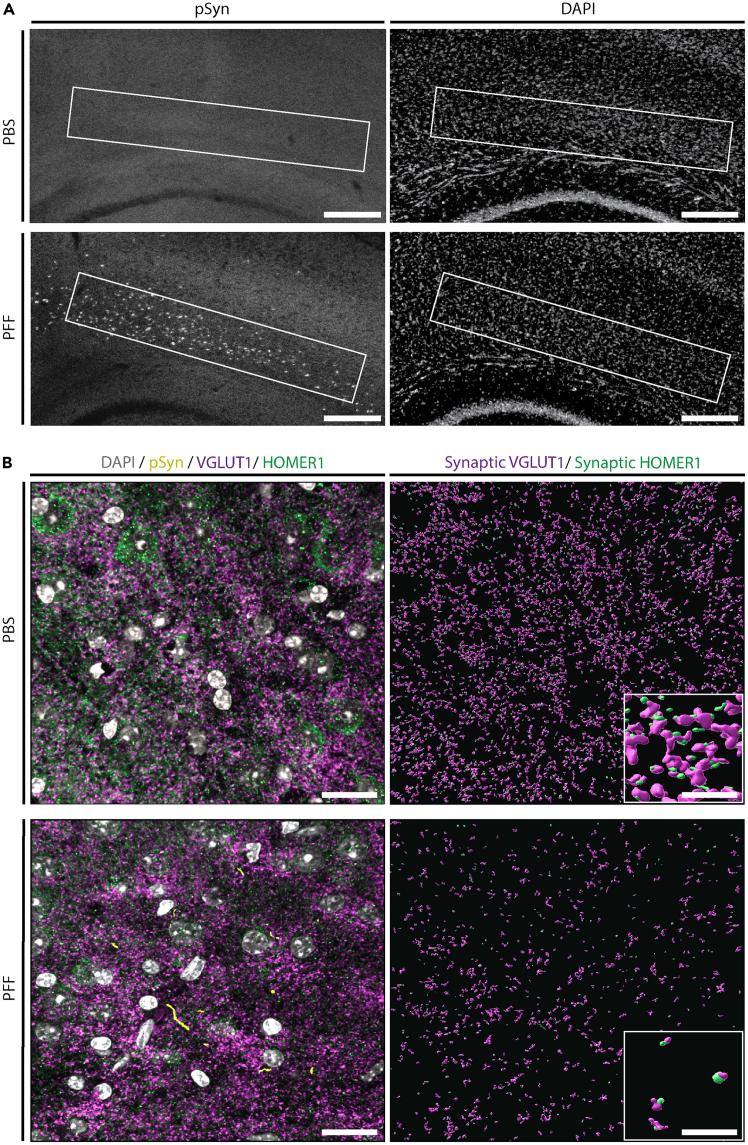
Synaptic surface rendering shows greater synaptic density reduction in PFF samples when compared to PBS samples (A) Low (4x) magnification representative acquired images for mice injected with preformed α-syn fibrils (PFFs) and mice that were injected with phosphate buffer saline (PBS). Upper panel (A) shows robust p-α-syn inclusions in PFF injected mice and lower panel (A) shows no p-α-syn inclusions in PBS treated mice. Using the SynDove protocol, we rendered surfaces for pre- and post-synaptic markers for PFF and PBS samples. (B) High (60x) magnification representative acquired images for mice injected with PFFs (p-α-syn inclusions highlighted in yellow) and mice that were injected with PBS. Scale bar = 200 µm and 15 µm for low magnification and high magnification, respectively.

Using this protocol, we confirmed excitatory synapse loss in C57BL/6J mice injected with either phosphate buffered saline (PBS) or alpha-synuclein pre-formed fibrils (PFF) 12 weeks post-injection (Figure 18). The region of interest (ROI) for this analysis was cortex with high pathological alpha-synuclein inclusion burden (Figure 18A). ‘Synaptic Density’ was quantified for VGLUT1 and HOMER1 per the instructions in “Statistical analysis for mean synaptic density and mean volume of puncta.” Using nested design, the synaptic density value (puncta/μm^3^) was calculated for 6 frames per animal (*n* = 3 per group) then analyzed via nested *t* test. These data show a reduction in VGLUT1/HOMER1+ synaptic density (Figures 18C and 18D).

We have also confirmed no loss of VGLUT1/HOMER1+ mean synaptic density in the amygdala of PFF-injected mice but rather increased mean volume of VGLUT1+ pre-synaptic terminals.^1^ Additionally, in the striatum of PFF-injected mice, we have confirmed a reduction in mean VGLUT1/HOMER1+ synaptic density compared to controls with a similar increase in volume of pre-synaptic terminals.^4^

## Quantification and statistical analysis

**Timing: 5 min (varies depending on the number of images)**

To perform a basic quantification of the density of synaptic loci within a frame, take the number of puncta determined by Imaris statistical analysis for the protein of interest. As an example, Imaris generates 2865 VGLUT2 puncta based on the previous surface generation parameters for one frame. This value can be divided by the total volume value of 34413.60 μm^3^ for the frame in which surfaces were generated. This calculation results in a density value of 0.0833 VGLUT2 puncta/μm^3^ per frame value.

$$\rho \left(VGLUT2\right)=\frac{VGLUT2\;count}{frame\;volume\;\left(\mu m^{3}\right)}=\frac{2865}{34413.60\;\mu m^{3}\;}=0.0833\;\mu m^{-3}\;VGLUT2\;per\;frame$$

Given the variability within and between experiments, it is advised to calculate the mean density of puncta across a minimum of four frames. As an example, the group mean for all frames can be calculated to determine the average VGLUT2 puncta per frame for each mouse.

$$\chi_{\rho }\left(VGLUT2\right)=\frac{\sum_{n}\;\rho_{VGLUT2\;\left(n\right)}\;}{n}=\frac{0.0833+0.0858+0.0789+0.0857\;}{4\;}=0.0834\;\mu m^{-3}\;mean\;density\;of\;VGLUT2\;for\;mouse\;X$$

Calculations can be repeated to determine the mean volume of VGLUT2 puncta within each image. For example, the mean volume for VGLUT2 across all VGLUT2 puncta within the frame is 0.5484 μm^3^. Note the equivalent measurement for all images from one animal, sum the values and divide by the total number of images collected for a group mean for VGLUT2 volume.

$$\chi_{vol}\left(VGLUT2\right)=\frac{\sum_{n}\;{vol}_{VGLUT2\;\left(n\right)}\;}{n}=\frac{0.5484+0.5834+0.4532+0.4656\;}{4\;}=0.5127\;\mu m^{3}\;mean\;vol\;of\;VGLUT2\;for\;mouse\;X$$

By applying the appropriate statistical tests through tools like R, Prism and SPSS, these values can be used to quantify changes in VGLUT2 density and mean volume of VGLUT2 to determine if there are differences between experimental conditions. It is our recommendation that sufficient time is spent devising the appropriate statistical analysis strategy to ensure the best experimental outcomes before beginning the analysis.

## Limitations

This method has been validated in fixed brain tissue. The parameters outlined in this protocol have been optimized for confocal microscopy images and synaptic proteins. High resolution images are needed for optimal use of this protocol. Super resolution techniques are recommended for better signal resolution. However, appropriate adjustments would need to be made to the above protocol for use with super resolution images. Alternatively, this protocol can also be used with expansion microscopy tissue to better resolve proteins.

## Troubleshooting

### Problem 1

File corruption as a result of image conversion to. IMS format. In order to generate surfaces using IMARIS, deconvolved images must be converted to the. IMS format (Figure 2). Occasionally, this conversion may result in file corruption or “streaking” (Related to ‘Convert Images to IMS Format’ > Step 2a).

### Potential solution

**Figure 19:**
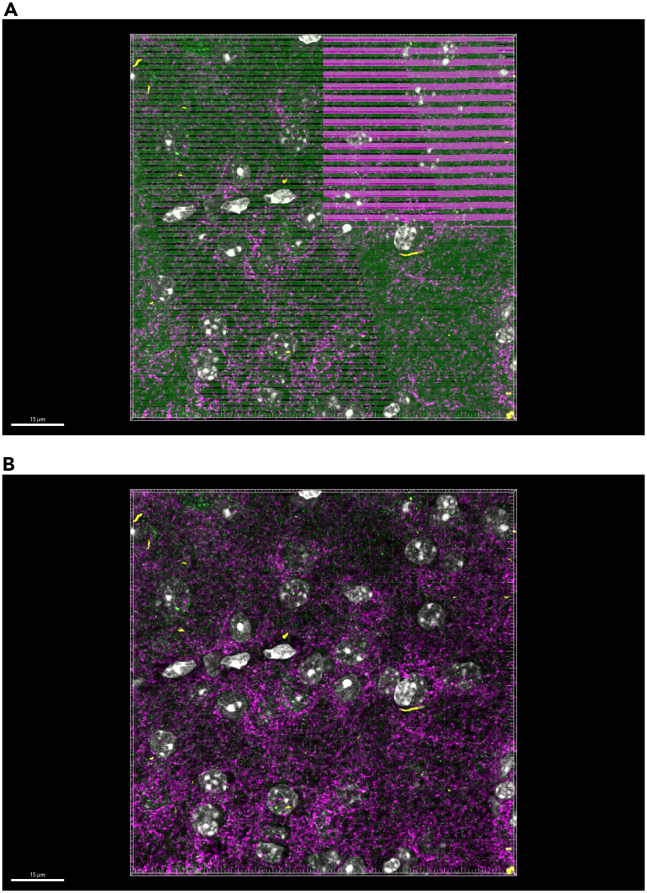
Resolving issue with file corruption (A) Corrupt file added to the input/output prompt in Imaris File Converter. (B) After selecting ‘Start All’ in software, a new file without corruption will automatically appear in Imaris Arena.

• Take note of corrupted file name and locate original deconvolved file. This file can be found in the Arena View (Figure 1A).
• Open Imaris File Converter and add original deconvolved file to input/output prompt.
• Select Start All. A new, uncorrupted image without streaking will automatically appear in Imaris Arena Figure 19.

### Problem 2

Incomplete acquisition of Z-stack due to slide movement. Slides that are not set in a fixed position can result in blurry z-stack images (Related to ‘turn on the confocal microscope’ > Step 2).

### Potential solution

• Stage clip placement: When mounting slide, ensure that stage clips are set in place. This will secure slide, when acquiring images that will be used for surface rendering.

### Problem 3

Inconsistent synapse density within the same brain layer (Related to ‘turn on the confocal microscope’ > Step 5).

### Potential solution

• Brain region variation: When planning imaging parameters, take note of specific layer ranges of the brain. When imaging, ensure that brain layers are consistent.

### Problem 4

False Surface Generation: False surface rendering due to the presence lipofuscin in samples. This could potentially lead to variations and impact statistical analysis (Related to ‘turn on the confocal microscope’ > Step 4).

### Potential solution

• Cupric Sulfate: When performing immunohistochemistry, it is advised to include a cupric sulfate wash (10-15 min) to resolve lipofuscin that may remain in the sample.

### Problem 5

Quality and detection of synaptic puncta: The quality and ability to detect synaptic puncta could be affected by upstream factors, including tissue fixation and tissue staining. The following are recommendations we have optimized, but other tissue collection, processing, and immunofluorescence protocols could be used (Related to ‘Deconvolve Images’ > Step 16).

### Potential solution

• For tissue collection, we recommend perfusing animals with 0.9% saline with heparin and sodium nitroprusside, followed by cold 4% paraformaldehyde.^1^
• For tissue sectioning, we recommend sectioning tissue at 40μm thickness.^1^
• For immunofluorescence, we recommend only including detergents (e.g., Triton-X) in blocking solutions. Primary and secondary antibody solutions should be detergent-free. We also recommend an antigen retrieval step, such as 10mM sodium citrate/0.05% Tween-20 (pH 6.0).^1^

### Problem 6

Unpaired surfaces vs. synaptic loci: The detection and quantification of synaptic loci will be limited by surfaces considered ‘unpaired,’ which is determined by the shortest-distance threshold (e.g., 0.01μm). ‘Unpaired surfaces’ may not truly be biologically unpaired, which could impact synaptic quantification. However, using a more constrained shortest-distance threshold is recommended to prevent the false detection of synapses potentially due to background noise or the spatial resolution limits of confocal microscopy (Related to ‘Isolate Surfaces for synaptic Proteins That are Closely Juxtaposed- Synaptic Loci’ > Step 31).

### Potential solution

• Synaptic clefts for among central synapses, neuromuscular junctions, and immune synapses can differ.^2^^,^^3^ To determine the upper threshold value for the shortest distance between pre-synaptic and post-synaptic puncta, review the literature related to the reported colocalization distances between synaptic proteins.
• Here is an example on how to determine the shortest-distance threshold, as referenced in Step 31a-i in the main protocol: Generate shortest distance surfaces for pre-synaptic protein to post-synaptic protein for values 0.02 μm to 0.001 μm in increments of 0.002 μm (Table 2). Repeat these steps for all control group images. Then, generate table for number of surfaces generated for each shortest distance step as a percentage of total surfaces for that protein (Table 2). Determine best cutoff value for shortest distance between synaptic proteins.

**Table 2:** The mean and median of these example shortest distance values equal 0.01 μm, which provides a conservative shortest-distance threshold

Shortest distance (μm)	VGLUT2 surfaces ‘synaptic’ (% of total VGLUT2 Population)	Homer1 surfaces ‘synaptic’ (% of total Homer1 Population)
0.020	45.47	56.43
0.018	45.24	56.08
0.016	45.03	55.72
0.014	44.80	55.32
0.012	44.63	55.01
0.010	44.31	54.62
0.008	44.13	54.27
0.006	43.83	53.83
0.004	43.62	53.47
0.002	43.27	53.00
0.001	43.16	52.86

**Figure 20:**
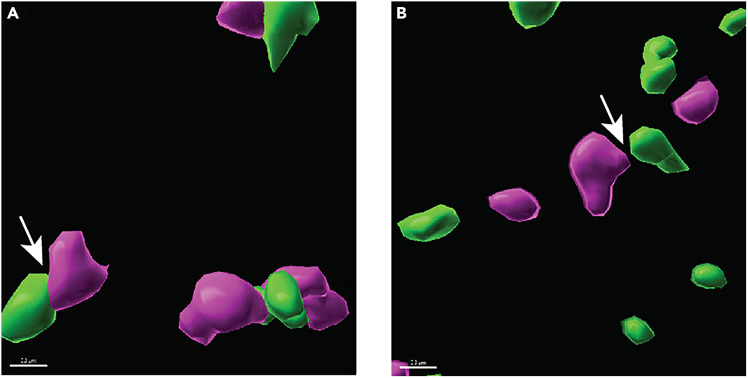
Comparison between closely juxtaposed and distant synaptic puncta Example images of pre- and post-synaptic puncta (A) closely juxtaposed using a shortest-distance threshold of 0.01μm and (B) distant from one another using a shortest-distance threshold of 0.02μm. Arrows indicate space between pre- and post-synaptic puncta. Scale bar = 10 µm.

• Based on the example provided (Table 2), using a shortest-distance threshold of 0.01 μm yielded pre- and -post-synaptic puncta that can be confirmed as closely juxtaposed (Figure 20A), whereas a shortest-distance threshold of 0.02 μm yielded synaptic loci that visually appear distant from one another (Figure 20B).

### Problem 7

Lack of access to Imaris software. The advantage of SynDOVE is that the three-dimensional structure of synapses is maintained allowing users to measure synaptic volumes. For example, we recently found that synapses harboring small alpha-synuclein aggregates show increased volumes.^1^ However, if the experimenter cannot access an Imaris software license, most of the concepts described in this protocol can be applied to alternative software programs. Examples of these alternative software programs are below.

### Potential solution

• SEQUIN requires Zeiss Airyscan, puncta detection using Imaris or Python and can measure distance between pre- and post-synaptic centroids.^5^
• SynBot is a method using the open-source Image J, which uses machine learning to threshold puncta corresponding to pre- and post-synaptic marker immunofluorescence, in addition to measuring the distance between pre- and post-synaptic markers.^6^

### Problem 8

The quality of puncta detection could be limited by image acquisition parameters (Related to ‘Setting up Imaging Parameters’ > Step 8).

### Potential solution

• Before surface generation, image acquisition must be optimized to ensure strong signal-to-noise ratio and minimizes photobleaching throughout the z-stack. Importantly, the parameters must be held constant within each image in a batch and must be optimized before data collection.
• Below is a table (Table 3) that summarizes the recommended minimum image acquisition parameters for puncta detection and surface segmentation during 3D surface rendering in Imaris. These parameters must be calibrated using pilot images and applied to the entire batch of images to be compared.

**Table 3:** Recommended image acquisition settings for puncta detection

Parameter	Recommended setting
Objective Lens	High-NA Oil Objective (e.g., 60x or 63x, NA ≥ 1.3)
Scan (x-y)	1024 x 1024 pixels or higher
Lateral Voxel (x-y)	0.10–0.12 μm
Axial Voxel (z)	Nyquist-compliant for objective
Scan Speed	Slow – moderate, optimize during pilot imaging and apply fixed value per batch
Pixel Dwell Time	≥ 1-2 μs, optimized during pilot imaging and apply fixed value per batch
Line/Frame Averaging	2-4, maintain low value and apply fixed value per batch
Laser Power & detector Gain	Maintain below saturation levels and apply fixed value per batch

## Resource availability

### Lead contact

Further information and requests for resources and reagents should be directed to and will be fulfilled by the lead contact, Laura A. Volpicelli-Daley (lvolpicellidaley@uabmc.edu).

### Technical contact

Questions about the technical specifics of performing the protocol should be directed to the technical contact, Arielle F. Manabat (amanabat@uab.edu) or Khaliah Y. Long (klonguab@uab.edu).

### Materials availability

This study did not generate new materials or reagents.

### Data and code availability

This study did not produce new code.

## Acknowledgments

The authors thank BioRender.com for providing the software used to create portions of the graphical abstract. This work was supported by the 10.13039/100000002National Institutes of Health (10.13039/100000065NINDS grant R56NS117465), Parkinson Association of Alabama (grant 977962), Aligning Science Across PD-Team Thomas Biederer (10.13039/100018231ASAP 020616) through the 10.13039/100000864Michael J Fox Foundation for Parkinson’s Research to L.A.V.-D., and Alzheimer’s of Central Alabama.

## Author contributions

N.Z.G. performed all sample preparation, collected data for this report, and wrote the manuscript. K.Y.L. and A.F.M. collected data for this report and edited the manuscript. L.A.V.-D. was responsible for overall project management and edited the manuscript.

## Declaration of interests

The authors declare no competing interests.
